# DNA Integrity of Chironomids and Oligochaetes Is Maintained During Long‐Term Storage in Neutral Buffered Formalin: Implications for Macroinvertebrate Monitoring

**DOI:** 10.1002/ece3.74047

**Published:** 2026-07-27

**Authors:** Régis Vivien, Patrick Martin, Michel Lafont, Benoît J. D. Ferrari

**Affiliations:** ^1^ Swiss Centre for Applied Ecotoxicology (Ecotox Centre) Eawag‐EPFL Lausanne/Dübendorf Switzerland; ^2^ Royal Belgian Institute of Natural Sciences, Taxonomy and Phylogeny Brussels Belgium; ^3^ Univ Lyon, Université Claude Bernard Lyon 1, CNRS, ENTPE, UMR 5023 LEHNA Villeurbanne France

**Keywords:** chironomids, DNA integrity, long‐term storage, macroinvertebrate monitoring, morphological preservation, neutral buffered formalin, oligochaetes

## Abstract

Oligochaetes and chironomids are valuable bioindicators but ethanol preservation is problematic: it causes oligochaetes to disintegrate and forces a trade‐off in insects between DNA integrity and morphological structures. Aiming to develop genetic oligochaete and chironomid indices based on the high‐throughput sequencing (HTS) of genetically tagged specimens, we tested whether long‐term specimen storage in neutral buffered formalin affects the success rate of subsequent genetic analyses. We performed PCR amplifications of the COI gene on specimens preserved in neutral buffered formalin for periods up to 6 months. For oligochaetes, DNA quantities (amplicon yields) decreased over time but remained sufficient for sequencing at all tested periods; for chironomids, no temporal trend toward degradation was observed. Storing these tissues in neutral buffered formalin for up to 6 months does not prevent subsequent genetic analyses and provides ample time for logistical processing, such as labor‐intensive sieving. These results facilitate the implementation of oligochaete and chironomid indices using a single sediment sample for both groups. More broadly, replacing ethanol with neutral buffered formalin as a fixative simultaneously preserves DNA quality, specimen density, community composition, and morphological structures across macroinvertebrate taxa. This integrated preservation allows for: (1) the refinement of macroinvertebrate indices both morphology‐ and bulk‐DNA‐based, by ensuring that all taxa, including soft‐bodied organisms, are accurately represented; (2) the perfection of insect barcode reference databases by enabling a dual validation approach where high‐quality sequences are directly linked to intact morphological vouchers; and (3) the improvement of quality control in bulk‐sample HTS by allowing morphological verification of molecular results on the same specimens.

## Graphical abstract

This study demonstrates that neutral buffered formalin effectively preserves both the morphological integrity and DNA quality of oligochaetes and chironomids for up to 6 months. By maintaining a stable pH, this fixation method overcomes the traditional ‘fixative dilemma’, allowing for reliable integrative taxonomy and long‐term DNA‐based monitoring.

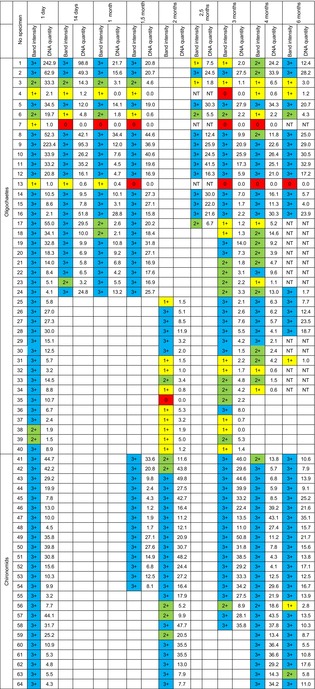

## 1 Introduction

Chironomids and oligochaetes are valuable indicators of sediment quality in rivers and lakes (Lafont et al. 2012; Saether 1979; Wiederholm 1980). Since morphological identification of these taxonomic groups at the species level is difficult and only possible for part of the specimens present in a sample (Carew et al. 2003; Vivien et al. 2017), indices based on genetic identification are being developed. High‐throughput sequencing (HTS) of samples composed of genetically tagged specimens (Shokralla et al. 2014; Gueuning et al. 2019; Vivien et al. 2020, 2023) has proven to be a promising way to identify the species present in a sample and estimate their relative abundance.

DNA is best preserved in absolute ethanol at −20°C. However, preservation of oligochaetes and chironomids (and insects in general) in ethanol presents significant logistical and biological limitations. Logistically, maintaining the high ethanol concentrations required for DNA integrity in bulky sediment samples necessitates excessive volumes of preservative to counteract dilution by interstitial water, significantly increasing the weight, cost, and complexity of sample transport. Biologically, in oligochaetes, the addition of ethanol to sediment samples can lead to fragmentation and disintegration of specimens, particularly when large amounts of sediments are present, resulting in biased estimates of abundance and diversity (Rodriguez and Reynoldson 2011). This phenomenon is likely related to the heterogeneity of ethanol concentration within the sediment, as individuals exposed to low ethanol concentrations remain susceptible to degradation. In addition, ethanol preservation makes specimens more fragile and therefore more prone to damage during sieving. It may also alter internal organs used for the morphological identification of oligochaetes, making this preservation method unsuitable when subsequent taxonomic work relies on physical examination. For chironomids, and insects in general, no single ethanol concentration optimally preserves both DNA and morphological structures. Preservation in 70% ethanol is adequate for preserving morphological structures but is suboptimal for DNA preservation because degradation may occur after a certain period of storage (1 year; Marquina et al. 2021). In contrast, absolute ethanol makes external structures brittle and prone to breakage, potentially causing the loss of diagnostic characters and impairing species‐level identification (Marquina et al. 2021). This high‐concentration preservative can also damage soft tissues, which may prevent taxonomic assignments based on the examination of internal features. Thus, the use of ethanol as a preservative entails a trade‐off between optimal DNA preservation and the maintenance of morphological characters.

On the other hand, formalin is recognized as suitable for preserving all organisms, including soft‐bodied invertebrates (Pfannkuche and Thiel 1988; Mackie 1994; Wilson 2005) and was widely used from the early 1900s–2010 for the fixation of organisms, including insects (AFNOR 2004; Holleley and Hahn 2025). It has been shown that aquatic oligochaetes fixed with formalin and subsequently stored in absolute ethanol do not suffer tissue damage (Vivien et al. 2018). This protection effectively extends to other macroinvertebrates, as formalin fixation provides a structural stabilization that shields specimens against the otherwise damaging effects of absolute ethanol. Conventional low‐pH formalin is not suitable when genetic analyses are subsequently performed, as it induces negative effects on DNA such as covalent cross‐linking, irreversible denaturation, modification, and fragmentation (Chaw et al. 1980; Tang 2006; Hykin et al. 2015). In fact, the pH of this fixative significantly impacts the success of DNA amplification and sequencing, with neutral buffered solutions being less damaging than low‐pH variants (Koshiba et al. 1993; Schander and Halanych 2003; Holleley and Hahn 2025). Furthermore, the amplification success rate tends to decline as storage duration increases (Schander and Halanych 2003; Bucklin and Allen 2004; Baird et al. 2011). While the adverse effects of low‐pH formalin are well‐documented, the precise temporal window for successful DNA recovery in neutral buffered formalin across different macroinvertebrate taxa remains poorly defined.

In previous efforts to develop a genetic oligochaete index, tests were conducted to determine the impact of formalin pH and storage duration on DNA quality (Vivien et al. 2016, 2018). While the success rate of COI fragment amplification and sequencing clearly decreased after only 3–7 days in low‐pH formalin, storage in a neutral buffered solution for up to 4 weeks had no negative effect. This approach has also proven effective for chironomids, as we were able to sequence several hundred chironomid specimens—belonging to a large number of species—preserved in neutral buffered formalin for up to 1 month without any difficulty (unpublished data). The four‐week period for storing oligochaete and chironomid tissues in formalin often allows sufficient time to perform sieving (the sieved material is then stored in absolute ethanol). However, this period may sometimes be insufficient, e.g., when a high number of samples are collected and/or if the sampling is carried out abroad. It would be necessary to be able to store sediment samples in neutral buffered formalin for more than 1 month, ideally for up to 4–6 months.

In this study, we assessed whether oligochaete and chironomid tissues preserved in neutral buffered formalin for up to 6 months remain suitable for genetic analyses, with the ultimate goal of supporting the development of genetic indices based on HTS of genetically tagged specimens. Specifically, we tested the extraction and amplification of a 658 bp fragment of the COI gene at different storage intervals. At the end of each period, PCR products were quantified and visualized on electrophoresis gels.

Although ethanol preservation is less damaging for chironomids than for oligochaetes, we included this group in our tests for three main reasons. First, analyzing both communities from a single sediment sample optimizes logistical workflows and significantly reduces field and laboratory costs. Second, using a single sample ensures data consistency and comparability, as biological quality can vary between separate samples at the same site due to micro‐habitat heterogeneity. Third, as previously noted, neutral buffered formalin potentially offers a unique solution to the trade‐off between DNA integrity and morphological preservation in insects, which ethanol cannot resolve.

## 2 Material and Methods

### 2.1 Sampling and Preparation of Samples

To collect oligochaetes, sediment samples were taken in December 2024 using a shovel in the Sorge River (Switzerland, Canton of Vaud) and placed in a 5‐L container. The oligochaetes were fixed in the field with a 4% formaldehyde solution prepared by a 5‐fold dilution of concentrated neutral buffered formalin (Epredia, Kalamazoo, MI, USA); the supernatant was removed before adding the fixative. Samples were then transported to the laboratory at room temperature, and sediments were immediately sieved. After sieving, oligochaetes were sorted and 40 specimens were placed individually in 1.5 mL Eppendorf tubes containing 400 μL of 4% neutral buffered formalin. At the following preservation periods, tissue samples were taken from each specimen, rinsed briefly with tap water in a Petri dish, and transferred to 1.5 mL Eppendorf tubes containing 0.3 mL of absolute ethanol, which were then stored at −20°C: 1 day (40 specimens), 14 days (24 specimens), 1 month (24), 1.5 months (24), 2 months (16), 2.5 months (14), 3 months (40), 4 months (34), and 6 months (21). The number of specimens analyzed at 4 and 6 months was reduced (*n* < 40), as several individuals had no remaining tissue after the previous testing intervals. Specimens were not identified to the species level, as only a stereomicroscope was used; they mainly belonged to Tubificinae and Lumbriculidae.

The chironomid material came from a culture of *Chironomus riparius* larvae. Twenty‐four specimens were fixed individually in 1.5 mL Eppendorf tubes containing 400 μL of 4% neutral buffered formalin. At the following periods, tissue fragments were extracted, rinsed briefly with tap water in a Petri dish, and transferred to 1.5 mL Eppendorf tubes containing 0.3 mL of absolute ethanol, which were then stored at −20°C: 1 day (24 specimens), 1.5 months (14 specimens), 2 months (24), 3 months (18), 4 months (24), and 6 months (24). All 24 chironomid specimens were available for analysis at 6 months.

Instead of being pre‐sampled, tissue fragments were collected from each oligochaete and chironomid specimen at each time point, resulting in potential size variations between periods. To minimize differences in starting material mass, fragment sizes were visually standardized across all sampling points, as achieving identical tissue mass was practically unfeasible. However, such variations are unlikely to limit PCR success. In fact, smaller fragments can often yield amplification results comparable to or even better than larger ones from the same individual.

All tissue fragments were air‐dried for 24 h prior to DNA extraction.

### 2.2 DNA Extraction, PCR and Quantification of PCR Products

Total genomic DNA was extracted from tissue samples using a modified version of the manual guanidine thiocyanate method described by Tkach and Pawlowski (1999). Briefly, tissue fragments were isolated and lysed in 100 μL of lysis buffer (containing 4 M guanidine thiocyanate, 0.1 M Tris–HCl pH 7.5, and 1% 2‐mercaptoethanol). Samples were incubated at 60°C for 16 h. After incubation, 100 μL of isopropanol was added to precipitate the DNA, and the mixtures were incubated at −20°C for at least 2 h. This was followed by centrifugation at 13,000 rpm for 20 min. The resulting DNA pellet was washed with 70% ethanol, centrifuged again at 13,000 rpm for 5 min, air‐dried to remove residual alcohol, and finally resuspended in 50 μL of Elution Buffer AE (Qiagen, Hilden, Germany). A 658 bp fragment of the COI gene was amplified using LCO1490 and HCO2198 primers (Folmer et al. 1994). Each PCR was performed in a total volume of 20 μL containing 0.2 μL of Taq polymerase 5 U/μL (Roche, Basel, Switzerland), 2 μL of the PCR buffer (10× concentrated) with MgCl_2_ (Roche), 0.5 μL of each primer (10 μM each), 0.4 μL of a 10 mM dNTP mix (Roche) and 1 μL of DNA template of undetermined concentration. PCR cycling conditions consisted of an initial denaturation step at 95°C for 5 min, followed by 35 cycles of denaturation at 95°C for 40 s, annealing at 44°C for 45 s and extension at 72°C for 1 min, with a final extension step at 72°C for 8 min.

PCR products were first examined on electrophoresis gels. PCR products showing bands of moderate to high intensity were considered suitable for sequencing by the Sanger method. PCR products corresponding to low‐intensity bands could also, in most cases, be successfully sequenced, provided that twice the amount of PCR products was used for sequencing. In addition, PCR products were quantified using the Qsep100 Capillary Electrophoresis System (BioOptic, New Taipei City, Taiwan) in order to verify DNA quality and to determine whether PCR amplification decreased over time and, if so, from which preservation period. The signal intensity of each amplicon was measured in Relative Fluorescence Units (RFU), a standard non‐absolute unit used to quantify DNA fluorescence during capillary migration. PCR products were diluted 1:11 in the buffer (kit) before analysis with the Qsep100 capillary electrophoresis system to ensure signal intensity remained within the linear range of the detector.

## 3 Results

### 3.1 Oligochaete Specimens

The visualization inspection of PCR products on electrophoresis gels revealed variations in band intensity over time, as shown by the intensity scores summarized in Table 1. Only two specimens (Nos. 7 and 13) could not be amplified after 1 day or 1 month, but the intensity of the bands corresponding to these specimens at 1 day was very low, indicating that these specimens likely posed DNA extraction problems for unknown reasons. Band intensity tended to decrease significantly (strong/medium to weak) over time for the following nine specimens: No. 3 at 2.5 months and after, No. 17 at 3 months and after, No. 23 at 4 months, and Nos. 31, 32, 34, 37, 38, and 40 at 2 months and after. For specimen No. 35, we observed no band at 2 months and a moderately intense band at 3 months, and for specimen No. 4, no band at 3 months and weak bands at 4 and 6 months. The amplification success rate was 100% at 1 day, 96% at 14 days and 1 month and between 92% and 95% at other times. Excluding the problematic specimens No. 7 and 13, the amplification success rate was 98–100% at all tested periods except at 2 months (94%). If specimens No. 4 and 35, whose amplification success rate does not appear to be related to the fixative (as the amplification success rate varies over time), are also excluded, the amplification success rate is 100% at all tested periods.

**TABLE 1:** Band intensities (electrophoresis gels) and DNA (PCR product) quantities (in ng/μL, undiluted values) obtained at each storage period (from 1 day to 6 months) of oligochaete (No. 1–40) and chironomid (No. 41–64) tissues in neutral buffered formalin. For band intensity, 3+ = strong intensity, 2+ = medium, 1+ = low, 0 = no visible band. The colors correspond to the different band intensities to facilitate reading (blue = strong, green = medium, yellow = low, red = no visible band). NT = Not tested.

	No specimen	1 day		14 days		1 month		1.5 month		2 months		2.5 months		3 months		4 months		6 months	
		Band intensity	DNA quantity	Band intensity	DNA quantity	Band intensity	DNA quantity	Band intensity	DNA quantity	Band intensity	DNA quantity	Band intensity	DNA quantity	Band intensity	DNA quantity	Band intensity	DNA quantity	Band intensity	DNA quantity
Oligochaetes	1	3+	242.9	3+	98.8	3+	21.7	3+	20.8			1+	7.5	1+	2.0	2+	24.2	3+	12.4
	2	3+	62.9	3+	49.3	3+	15.6	3+	20.7			3+	24.5	3+	27.5	2+	33.9	3+	28.2
	3	2+	33.3	2+	14.3	2+	3.1	2+	4.6			1+	1.8	1+	1.1	1+	6.5	1+	3.0
	4	1+	2.1	1+	1.2	1+	0.0	1+	0.0			NT	NT	0	0.0	1+	0.6	1+	1.2
	5	3+	34.5	3+	12.0	3+	14.1	3+	19.0			3+	30.3	3+	27.9	3+	34.3	3+	20.7
	6	2+	19.7	1+	4.8	2+	1.8	1+	0.6			2+	5.5	2+	2.2	1+	2.2	2+	4.3
	7	1+	1.0	0	0.0	0	0.0	0	0.0			NT	NT	0	0.0	0	0.0	NT	NT
	8	3+	52.3	3+	42.1	3+	34.4	3+	44.6			3+	12.4	3+	9.9	2+	11.8	3+	25.0
	9	3+	223.4	3+	95.3	3+	12.0	3+	36.9			3+	25.9	3+	20.9	3+	22.6	3+	29.0
	10	3+	33.9	3+	26.2	3+	7.6	3+	40.6			3+	24.5	3+	25.9	3+	26.4	3+	30.5
	11	3+	33.2	3+	35.2	3+	4.5	3+	19.6			3+	41.5	3+	17.3	3+	25.1	3+	32.9
	12	3+	20.8	3+	16.1	3+	4.7	3+	16.9			3+	16.3	3+	5.9	3+	21.0	3+	17.2
	13	1+	1.0	1+	0.6	1+	0.4	0	0.0			NT	NT	0	0.0	0	0.0	0	0.0
	14	3+	10.5	3+	9.5	3+	10.1	3+	27.3			3+	30.0	3+	7.0	3+	16.1	3+	5.7
	15	3+	8.6	3+	7.8	3+	3.1	3+	27.1			3+	22.0	3+	1.7	3+	11.3	3+	4.0
	16	3+	2.1	3+	51.8	3+	28.8	3+	15.8			3+	21.6	3+	2.2	3+	30.3	3+	23.9
	17	3+	55.0	3+	29.5	2+	2.6	3+	20.2			2+	6.7	1+	1.2	1+	5.2	NT	NT
	18	3+	34.1	3+	10.0	2+	2.1	3+	18.4					1+	1.3	2+	14.6	NT	NT
	19	3+	32.8	3+	9.9	3+	10.8	3+	31.8					3+	14.0	2+	9.2	NT	NT
	20	3+	18.3	3+	6.9	3+	9.2	3+	27.1					3+	7.3	2+	3.9	NT	NT
	21	3+	14.0	3+	5.8	3+	6.8	3+	16.9					2+	1.8	2+	4.7	NT	NT
	22	3+	8.4	3+	6.5	3+	4.2	3+	17.6					2+	3.1	3+	9.6	NT	NT
	23	3+	5.1	2+	3.2	3+	5.5	3+	16.9					2+	2.2	1+	1.1	NT	NT
	24	3+	4.1	3+	24.8	3+	13.2	3+	25.7					2+	3.3	2+	13.0	3+	1.7
	25	3+	5.8							1+	1.5			3+	2.1	3+	6.3	3+	7.7
	26	3+	27.0							3+	5.1			3+	2.6	3+	6.2	3+	12.4
	27	3+	27.3							3+	8.5			3+	7.6	3+	5.7	3+	23.5
	28	3+	30.0							3+	11.9			3+	5.5	3+	4.1	3+	18.7
	29	3+	15.1							3+	3.2			3+	4.2	3+	2.1	NT	NT
	30	3+	12.5							3+	2.0			3+	1.5	2+	2.4	NT	NT
	31	3+	5.7							1+	1.5			1+	2.2	2+	4.2	1+	1.0
	32	3+	3.2							1+	1.0			1+	1.7	1+	0.6	NT	NT
	33	3+	14.5							2+	3.4			2+	4.8	2+	1.5	NT	NT
	34	3+	8.8							1+	0.8			2+	4.2	1+	0.6	NT	NT
	35	3+	10.7							0	0.0			2+	2.2				
	36	3+	6.7							1+	5.3			3+	8.0				
	37	3+	2.4							1+	3.2			1+	0.7				
	38	2+	1.9							1+	1.9			1+	0.0				
	39	2+	1.5							1+	5.0			2+	5.3				
	40	3+	8.9							1+	1.2			1+	1.4				
Chironomids	41	3+	44.7					3+	33.6	2+	11.6			3+	46.0	2+	13.8	3+	10.6
	42	3+	42.2					3+	20.8	2+	43.8			3+	29.6	3+	5.7	3+	7.9
	43	3+	29.2					3+	9.8	3+	49.8			3+	44.6	3+	6.8	3+	13.9
	44	3+	19.9					3+	2.4	3+	27.5			3+	39.9	3+	5.9	3+	9.1
	45	3+	7.8					3+	4.3	3+	42.7			3+	33.2	3+	8.5	3+	25.2
	46	3+	13.0					3+	1.2	3+	16.4			3+	22.4	3+	39.2	3+	21.6
	47	3+	10.0					3+	1.9	3+	11.2			3+	13.5	3+	43.1	3+	35.1
	48	3+	4.5					3+	1.7	3+	12.1			3+	11.0	3+	27.4	3+	15.7
	49	3+	35.8					3+	27.1	3+	20.9			3+	50.8	3+	11.2	3+	21.7
	50	3+	39.8					3+	27.6	3+	30.7			3+	31.8	3+	7.8	3+	15.6
	51	3+	30.8					3+	14.9	3+	48.2			3+	38.5	3+	4.3	3+	13.8
	52	3+	15.6					3+	6.8	3+	24.4			3+	29.2	3+	4.1	3+	17.1
	53	3+	10.3					3+	12.5	3+	27.2			3+	33.3	3+	12.5	3+	12.5
	54	3+	9.9					3+	8.1	3+	16.4			3+	34.2	3+	29.6	3+	16.7
	55	3+	3.2							3+	17.9			3+	27.5	3+	21.9	3+	13.9
	56	3+	7.7							2+	5.2			2+	8.9	2+	18.6	1+	2.8
	57	3+	44.1							2+	9.9			3+	28.1	3+	43.5	3+	13.5
	58	3+	31.7							3+	47.7			3+	35.8	3+	37.8	3+	10.3
	59	3+	25.2							2+	20.5					3+	13.4	3+	8.7
	60	3+	10.9							3+	35.5					3+	36.4	3+	5.5
	61	3+	5.3							3+	35.5					3+	36.6	3+	10.8
	62	3+	4.8							3+	13.0					3+	29.2	3+	17.6
	63	3+	5.5							3+	7.9					3+	14.3	2+	5.8
	64	3+	4.3							3+	7.7					3+	34.2	3+	11.0

Compared to the results obtained at 1 day, we observed a significant decrease in DNA quantity (amplicon yield) at 3–6 months for 27 oligochaete specimens (Table 1, Table S1). For 13 specimens, we obtained more or almost as much DNA at 3, 4, or 6 months than at 1 day. Significant amounts of DNA (> 0.5 ng/μL) were measured for 32 (out of 34) specimens and for 20 (out of 21) specimens at 4 and 6 months of storage, respectively. RFU analysis, performed exclusively on successfully amplified samples to focus on DNA quality trends, showed a global decrease in signal intensity, with mean intensities lower at 2–6 months than at 1 day (Table 2). However, RFU values remained globally high at 4 to 6 months; the median at 6 months (151.3) was even higher than the reference value at 1 day (128.8). This higher median at 6 months is partly explained by the fact that problematic specimens with very low initial signals (e.g., Nos. 7 and 13) were not amplified at later stages and were thus excluded from the RFU calculation. The lower median intensities recorded at 2 and 3 months (26.7 and 30.7 RFU, respectively) likely reflect individual sample variability or extraction yields rather than preservative‐induced degradation, given the recovery of high signals at 4 and 6 months. For visual assessment, we provide as Figure S1 representative electropherograms (specimen no. 11) at 1 day and 6 months showing high signal‐to‐noise ratio.

**TABLE 2:** Minimum, maximum, mean, and median values of relative fluorescence units (RFU, undiluted values) obtained for each storage period (from 1 day to 6 months), oligochaetes and chironomids separately. The total number of specimens corresponding to each storage period is indicated (*n*).

	RFU								
	1 day	14 days	1 month	1.5 months	2 months	2.5 months	3 months	4 months	6 months
*Oligochaetes*									
*n*	40	23	22	21	15	14	36	32	20
Min	7.9	5.3	3.7	4.5	6.2	15.7	2.4	4.5	7.8
Max	1898.6	1082.8	387.9	531.6	136.2	442.0	299.1	378.0	353.8
Mean	272.7	267.0	100.9	237.6	36.3	202.7	68.3	121.1	162.1
Median	128.8	126.6	75.1	204.9	26.7	225.4	30.7	62.6	151.3
*Chironomids*									
*n*	24			14	24		18	24	24
Min	28.4			10.2	43.0		79.4	36.3	25.4
Max	531.7			384.0	561.9		585.2	504.4	364.7
Mean	206.9			131.1	262.8		334.1	233.3	143.2
Median	117.4			88.4	217.6		352.6	181.2	132.2

### 3.2 Chironomid Specimens

We observed no trend toward a decrease in PCR band intensity and DNA quantities (amplicon yields) over time (Table 1, Table S1). At 6 months, the amplification success rate remained 100%. RFU values remained remarkably consistent, with a median at 6 months (132.2) slightly higher than the reference value at 1 day (117.4) (Table 2). We also provide as Figure S1 the electropherograms of a chironomid specimen (specimen no. 52) at 1 day and 6 months.

## 4 Discussion

Our study shows that storing oligochaete and chironomid tissues in neutral buffered formalin for up to 6 months does not prevent subsequent genetic analyses. These results suggest that DNA degradation typically associated with formalin is primarily driven by low‐pH acidity rather than by the formaldehyde molecules themselves. We observed a general trend toward a decrease in PCR band intensity and DNA quantities (amplicon yields) over time for oligochaetes, but not for chironomids. Possible causes for this decrease include the effect of formaldehyde itself, but also variations in the amount of tissue extracted. However, for almost all specimens, significant amounts of DNA and bands of sufficient intensity for sequencing were obtained at all preservation periods.

The observed difference between the two groups suggests a taxonomic variation in sensitivity to long‐term neutral buffered formalin storage. While low pH is the primary driver of DNA degradation, formaldehyde molecules in a neutral solution may still induce minor, slow‐acting effects on DNA. In this context, it could be hypothesized that the chitinous cuticle of chironomids acts as a more effective physical barrier than the permeable integument of oligochaetes, limiting the rate at which formaldehyde interacts with the tissues.

If neutral buffered formalin induces DNA damage, it appears to be minimal. Previously, the COI, ITS2, and 28S markers of numerous specimens from various species stored in neutral buffered formalin for up to 1 month were successfully sequenced (Vivien et al. 2020, 2023, 2024). Furthermore, neutral buffered formalin storage for up to 6 months is suitable for amplifying smaller COI fragments than the 658 bp (Leray et al. 2013) commonly used for HTS of genetically tagged specimens (Vivien et al. 2020). Importantly, this 6‐month window provides ample time for the logistical processing of large sample volumes, including labor‐intensive sieving steps, before final transfer to ethanol. Finally, we emphasize that neutral buffered formalin should contain a maximum of 4% formaldehyde to ensure successful amplification, following the procedure proposed in Vivien et al. (2018).

These findings challenge the long‐standing assumption that formaldehyde is inherently incompatible with DNA‐based monitoring. By demonstrating that DNA degradation is primarily a function of low pH rather than the formaldehyde molecule itself, we provide a technical pathway to overcome the ‘fixative dilemma’. For oligochaetes, while neutral buffered formalin was already known to prevent specimen disintegration and morphological damage—common issues with ethanol—our results confirm that it also maintains DNA integrity for long periods. This validates neutral buffered formalin as a total fixative for this group, ensuring that specimen density and taxonomic resolution can be preserved without sacrificing molecular accessibility. A particularly important application concerns insects such as chironomids. Previously, researchers often had to choose between preserving fragile diagnostic structures and maintaining DNA quality, as standard ethanol concentrations can fail to meet both requirements simultaneously. By stabilizing both internal and external structures without compromising DNA amplification, neutral buffered formalin allows for the preservation of high‐quality vouchers where the physical specimen remains intact alongside its genetic information. This is paramount for building reliable barcode reference databases (via Sanger or HTS), as it maintains the essential link between high‐quality sequences and perfectly preserved morphological vouchers.

On a logistical level, our approach eliminates the need for separate sampling and sieving efforts by allowing the use of a single sediment sample for both oligochaetes and chironomids. Since neutral buffered formalin effectively preserves DNA quality in both soft‐bodied and chitinous taxa, it is likely adequate for preserving DNA quality across all macroinvertebrate groups. By simultaneously preserving DNA quality, specimen density, community composition, and morphological structures, neutral buffered formalin emerges as a superior tool for integrated ecological research. Beyond these specific indicators, this method could refine both traditional morphology‐based indices and current bulk‐sample HTS approaches across all macroinvertebrates (Krogmann and Holstein 2010; Elbrecht et al. 2017) by ensuring that all organisms, including soft‐bodied taxa, are accurately represented without the loss of taxonomic resolution. In addition, for biomonitoring based on bulk‐sample HTS, this method allows for non‐destructive or tissue‐subsampling approaches where researchers can perform molecular analyses while keeping the physical specimens available for morphological verification—a level of quality control previously hindered by the damaging effects of high‐concentration ethanol.

These findings help reconsider why ethanol replaced formalin in macroinvertebrate studies. While this shift was motivated by DNA preservation issues and toxicity concerns, we demonstrate that a neutral buffer resolves the former, while appropriate precautions, such as wearing a mask in the field and sieving sediment samples under a fume hood (Figure S2), effectively manage the latter. By ensuring a safe working environment and high‐quality data, neutral buffered formalin remains a viable and reliable tool for modern integrated taxonomy.

In conclusion, our results provide the technical foundation for implementing genetic oligochaete and chironomid indices based on HTS of genetically tagged specimens. By allowing the use of a single sediment sample for both groups and removing the pressure for rapid sieving, this approach significantly optimizes logistical workflows. Beyond these specific groups, this work offers broader implications for the field of biomonitoring by enabling: (1) the refinement of macroinvertebrate indices through the accurate representation of all taxa, including soft‐bodied taxa; (2) the perfection of insect reference databases by maintaining the link between high‐quality sequences and intact vouchers; and (3) a more reliable quality control framework in bulk‐sample HTS. While the use of formaldehyde requires rigorous safety protocols, it currently remains the only reliable tool for ensuring such high‐quality integration of taxonomic and genetic data. In the absence of an equally effective fixative, neutral buffered formalin should be considered a necessary transitional solution until a safer substitute offering equivalent technical performance is validated and made accessible to all practitioners. Consequently, future research into formaldehyde substitutes, particularly those emerging in the medical field (Bussolati et al. 2017; Sarot et al. 2017), must be intensified to eventually provide a monitoring framework for macroinvertebrates that is at once safe, sustainable, and scientifically robust.

## Author Contributions

**Régis Vivien:** conceptualization (lead), formal analysis (lead), investigation (lead), methodology (lead), writing – original draft (lead), writing – review and editing (lead). **Benoît J. D. Ferrari:** conceptualization (equal), formal analysis (supporting), investigation (supporting), methodology (equal), writing – original draft (supporting), writing – review and editing (supporting). **Patrick Martin:** conceptualization (supporting), formal analysis (equal), investigation (equal), methodology (supporting), writing – original draft (equal), writing – review and editing (equal). **Michel Lafont:** conceptualization (supporting), formal analysis (supporting), investigation (supporting), methodology (supporting), writing – original draft (equal), writing – review and editing (supporting).

## Funding

The authors have nothing to report.

## Conflicts of Interest

The authors declare no conflicts of interest.

## Supporting information


**Table S1:** Length of the amplified COI fragment (in base pairs), DNA (PCR product) concentration (in ng/μL, undiluted value), and relative fluorescence unit (RFU, undiluted value) for each oligochaete (no. 1–40) and chironomid (no. 41–64) specimen at each storage period (Neg. ctrl = negative control).
**Figure S1:** Representative COI amplicon profiles (electropherograms) obtained via Qsep100 capillary electrophoresis. (a) Oligochaete specimen (No. 11) at 1 day of storage; (b) Same oligochaete specimen at 6 months; (c) Chironomid specimen (No. 52) at 1 day; (d) Same chironomid specimen at 6 months.
**Figure S2:** Photo of a sample sieving setup (consisting of a basin and showerhead) installed in a fume hood.

## Data Availability

The data generated and analyzed during the current study are included in the article (Table 1, Table S1).
