# Supplementary material for: DNA Integrity of Chironomids and Oligochaetes Is Maintained During Long‐Term Storage in Neutral Buffered Formalin: Implications for Macroinvertebrate Monitoring
Source: Ecol Evol. 2026 Jul 27;16(7):e74047. doi: 10.1002/ece3.74047 (PMC13402932; doi:10.1002/ece3.74047)
Supplement: Supplementary file 1 — Table S1: Length of the amplified COI fragment (in base pairs), DNA (PCR product) concentration (in ng/μL, undiluted value), and relative fluorescence unit (RFU, undiluted value) for each oligochaete (no. 1–40) and chironomid (no. 41–64) specimen at each storage period (Neg. ctrl = negative control). Figure S1: Representative COI amplicon profiles (electropherograms) obtained via Qsep100 capillary electrophoresis. (a) Oligochaete specimen (No. 11) at 1 day of storage; (b) Same oligochaete specimen at 6 months; (c) Chironomid specimen (No. 52) at 1 day; (d) Same chironomid specimen at 6 months. Figure S2: Photo of a sample sieving setup (consisting of a basin and showerhead) installed in a fume hood. [file ECE3-16-e74047-s001.pdf]

DNA integrity of chironomids and oligochaetes is maintained during long-term storage in neutral buffered formalin: Implications for macroinvertebrate monitoring

Supplemental information

Table S1: Length of the amplified COI fragment (in base pairs), DNA (PCR product) concentration (in ng/µL, undiluted value), and relative fluorescence unit (RFU, undiluted value) for each oligochaete (no. 1–40) and chironomid (no. 41–64) specimen at each storage period. (Neg. ctrl = negative control)

Sample Name	Taxonomic group	No specimen	Storage period	Length (base pairs)	DNA concentration	RFU
1	oligochaete	1	1 day	597	242.88	1898.6
2	oligochaete	2	1 day	666	62.92	722.3
3	oligochaete	3	1 day	690	33.33	369.6
4	oligochaete	4	1 day	719	2.09	20.5
5	oligochaete	5	1 day	708	34.54	349.1
6	oligochaete	6	1 day	728	19.69	200.5
7	oligochaete	7	1 day	777	0.99	7.9
8	oligochaete	8	1 day	665	52.25	574.2
9	oligochaete	9	1 day	575	223.41	1844.4
10	oligochaete	10	1 day	688	33.88	383.8
11	oligochaete	11	1 day	719	33.22	337.3
12	oligochaete	12	1 day	718	20.79	217.7
13	oligochaete	13	1 day	732	0.99	10.2
14	oligochaete	14	1 day	734	10.45	102.6
15	oligochaete	15	1 day	735	8.58	80.6
16	oligochaete	16	1 day	749	2.09	18.9
17	oligochaete	17	1 day	629	55	687.4
18	oligochaete	18	1 day	703	34.1	369.9
19	oligochaete	19	1 day	694	32.78	370.6
20	oligochaete	20	1 day	718	18.26	196.0
21	oligochaete	21	1 day	732	13.97	139.2
22	oligochaete	22	1 day	742	8.36	78.7
23	oligochaete	23	1 day	748	5.06	45.9
24	oligochaete	24	1 day	745	4.07	37.1
25		Neg. ctrl			0	0.0
26	oligochaete	1	14 days	618	98.78	1066.0
27	oligochaete	2	14 days	701	49.28	524.8
28	oligochaete	3	14 days	748	14.3	139.5
29	oligochaete	4	14 days	728	1.21	13.4

Sample Name	Taxonomic group	No specimen	Storage period	Length (base pairs)	DNA concentration	RFU
30	oligochaete	5	14 days	726	11.99	126.6
31	oligochaete	6	14 days	762	4.84	42.8
32	oligochaete	7	14 days		0	0.0
33	oligochaete	8	14 days	681	42.13	494.1
34	oligochaete	9	14 days	615	95.26	1082.8
35	oligochaete	10	14 days	705	26.18	290.5
36	oligochaete	11	14 days	717	35.2	377.2
37	oligochaete	12	14 days	731	16.06	165.7
38	oligochaete	13	14 days	741	0.55	5.3
39	oligochaete	14	14 days	744	9.46	93.4
40	oligochaete	15	14 days	745	7.81	77.3
41	oligochaete	16	14 days	679	51.81	593.0
42	oligochaete	17	14 days	679	29.48	358.8
43	oligochaete	18	14 days	750	10.01	100.2
44	oligochaete	19	14 days	736	9.9	102.1
45	oligochaete	20	14 days	746	6.93	70.0
46	oligochaete	21	14 days	745	5.83	53.2
47	oligochaete	22	14 days	746	6.49	60.0
48	oligochaete	23	14 days	753	3.19	29.6
49	oligochaete	24	14 days	708	24.75	275.4
50	oligochaete	1	1 m	729	21.67	222.5
51	oligochaete	2	1 m	753	15.62	150.7
52	oligochaete	3	1 m	771	3.08	28.4
53	oligochaete	4	1 m		0	0.0
54	oligochaete	5	1 m	726	14.08	146.1
55	oligochaete	6	1 m	775	1.76	15.3
56	oligochaete	7	1 m		0	0.0
57	oligochaete	8	1 m	690	34.43	387.9
58	oligochaete	9	1 m	731	11.99	122.7
59	oligochaete	10	1 m	727	7.59	82.9
60	oligochaete	11	1 m	773	4.51	41.8
61	oligochaete	12	1 m	743	4.73	47.3
62	oligochaete	13	1 m	742	0.44	3.7
63	oligochaete	14	1 m	742	10.12	96.0
64	oligochaete	15	1 m	757	3.08	28.6
65	oligochaete	16	1 m	712	28.82	304.2
66	oligochaete	17	1 m	734	2.64	27.0
67	oligochaete	18	1 m	769	2.09	19.5
68	oligochaete	19	1 m	740	10.78	107.7
69	oligochaete	20	1 m	740	9.24	93.1
70	oligochaete	21	1 m	751	6.82	67.2

Sample Name	Taxonomic group	No specimen	Storage period	Length (base pairs)	DNA concentration	RFU
71	oligochaete	22	1 m	756	4.18	40.9
72	oligochaete	23	1 m	748	5.5	54.8
73	oligochaete	24	1 m	728	13.2	131.3
74	oligochaete	1	1.5m	734	20.79	212.3
75	oligochaete	2	1.5m	746	20.68	204.9
76	oligochaete	3	1.5m	770	4.62	43.1
77	oligochaete	4	1.5m		0	0.0
78	oligochaete	5	1.5m	717	19.03	193.9
79	oligochaete	6	1.5m	782	0.55	4.5
80	oligochaete	7	1.5m		0	0.0
81	oligochaete	8	1.5m	674	44.55	531.6
82	oligochaete	9	1.5m	689	36.85	408.1
83	oligochaete	10	1.5m	686	40.59	454.5
84	oligochaete	11	1.5m	746	19.58	196.8
85	oligochaete	12	1.5m	727	16.94	170.7
86	oligochaete	13	1.5m		0	0.0
87	oligochaete	14	1.5m	713	27.28	282.9
88	oligochaete	15	1.5m	715	27.06	277.1
89	oligochaete	16	1.5m	722	15.84	170.0
90	oligochaete	17	1.5m	693	20.24	241.5
91	oligochaete	18	1.5m	733	18.37	185.0
92	oligochaete	19	1.5m	700	31.79	339.5
93	oligochaete	20	1.5m	714	27.06	279.1
94	oligochaete	21	1.5m	726	16.94	168.2
95	oligochaete	22	1.5m	730	17.6	176.6
96	oligochaete	23	1.5m	732	16.94	163.7
97	oligochaete	24	1.5m	702	25.74	284.8
98	oligochaete	1	2.5m	750	7.48	73.5
99	oligochaete	2	2.5m	737	24.53	246.1
100	oligochaete	3	2.5m	769	1.76	15.7
101	oligochaete	5	2.5m	701	30.25	323.7
102	oligochaete	6	2.5m	764	5.5	51.7
103	oligochaete	8	2.5m	725	12.43	126.1
104	oligochaete	9	2.5m	715	25.85	271.0
105	oligochaete	10	2.5m	701	24.53	273.5
106	oligochaete	11	2.5m	708	41.47	442.0
107	oligochaete	12	2.5m	728	16.28	167.4
108	oligochaete	14	2.5m	704	30.03	325.5
109	oligochaete	15	2.5m	722	22	229.9
110	oligochaete	16	2.5m	723	21.56	220.9
111	oligochaete	17	2.5m	729	6.71	70.6
112		Neg. ctrl			0	0.0
113	oligochaete	1	3m	722	1.98	19.6

Sample Name	Taxonomic group	No specimen	Storage period	Length (base pairs)	DNA concentration	RFU
114	oligochaete	2	3m	732	27.5	279.6
115	oligochaete	3	3m	775	1.1	9.8
116	oligochaete	4	3m		0	0.0
117	oligochaete	5	3m	699	27.94	299.1
118	oligochaete	6	3m	768	2.2	20.4
119	oligochaete	7	3m		0	0.0
120	oligochaete	8	3m	727	9.9	100.1
121	oligochaete	9	3m	711	20.9	223.7
122	oligochaete	10	3m	704	25.85	286.3
123	oligochaete	11	3m	745	17.27	172.0
124	oligochaete	12	3m	747	5.94	61.2
125	oligochaete	13	3m		0	0.0
126	oligochaete	14	3m	749	7.04	68.4
127	oligochaete	15	3m	760	1.65	16.0
128	oligochaete	16	3m	754	2.2	20.4
129	oligochaete	17	3m	724	1.21	14.1
130	oligochaete	18	3m	759	1.32	12.7
131	oligochaete	19	3m	726	13.97	154.6
132	oligochaete	20	3m	742	7.26	77.8
133	oligochaete	21	3m	751	1.76	17.4
134	oligochaete	22	3m	756	3.08	29.9
135	oligochaete	23	3m	759	2.2	21.6
136	oligochaete	24	3m	760	3.3	31.6
137		Neg. ctrl			0	0.0
138	oligochaete	1	4m	721	24.2	266.5
139	oligochaete	2	4m	724	33.88	367.2
140	oligochaete	3	4m	761	6.49	63.0
141	oligochaete	4	4m	733	0.55	4.8
142	oligochaete	5	4m	695	34.32	378.0
143	oligochaete	6	4m	740	2.2	19.3
144	oligochaete	7	4m		0	0.0
145	oligochaete	8	4m	719	11.77	132.7
146	oligochaete	9	4m	709	22.55	265.9
147	oligochaete	10	4m	703	26.4	298.9
148	oligochaete	11	4m	735	25.08	268.7
149	oligochaete	12	4m	723	21.01	219.0
150	oligochaete	13	4m		0	0.0
151	oligochaete	14	4m	734	16.06	158.2
152	oligochaete	15	4m	744	11.33	109.8
153	oligochaete	16	4m	706	30.25	344.6
154	oligochaete	17	4m	720	5.17	60.3
155	oligochaete	18	4m	739	14.63	150.0
156	oligochaete	19	4m	739	9.24	97.0

Sample Name	Taxonomic group	No specimen	Storage period	Length (base pairs)	DNA concentration	RFU
157	oligochaete	20	4m	747	3.85	37.6
158	oligochaete	21	4m	749	4.73	46.8
159	oligochaete	22	4m	733	9.57	96.1
160	oligochaete	23	4m	766	1.1	9.4
161	oligochaete	24	4m	724	12.98	147.7
162	oligochaete	Neg. ctrl			0	0.0
163	oligochaete	1	6m	747	12.43	123.6
164	oligochaete	2	6m	737	28.16	289.1
165	oligochaete	3	6m	769	2.97	27.7
166	oligochaete	4	6m	731	1.21	12.4
167	oligochaete	5	6m	711	20.68	214.7
168	oligochaete	6	6m	765	4.29	36.5
169	oligochaete	8	6m	699	24.97	294.3
170	oligochaete	9	6m	702	29.04	329.6
171	oligochaete	10	6m	697	30.47	343.2
172	oligochaete	11	6m	720	32.89	353.8
173	oligochaete	12	6m	725	17.16	174.6
174	oligochaete	13	6m		0	0.0
175	oligochaete	14	6m	745	5.72	52.5
176	oligochaete	15	6m	751	3.96	36.2
177	oligochaete	16	6m	714	23.87	267.3
178	oligochaete	24	6m	762	1.65	16.0
179		Neg. ctrl			0	0.0
180	oligochaete	25	1 day	777	5.83	49.1
181	oligochaete	26	1 day	695	26.95	298.2
182	oligochaete	27	1 day	678	27.28	307.8
183	oligochaete	28	1 day	690	30.03	327.3
184	oligochaete	29	1 day	734	15.07	148.1
185	oligochaete	30	1 day	740	12.54	118.4
186	oligochaete	31	1 day	759	5.72	49.7
187	oligochaete	32	1 day	764	3.19	27.2
188	oligochaete	33	1 day	742	14.52	141.4
189	oligochaete	34	1 day	784	8.8	75.7
190	oligochaete	35	1 day	736	10.67	110.7
191	oligochaete	36	1 day	738	6.71	62.8
192	oligochaete	37	1 day	752	2.42	22.8
193	oligochaete	38	1 day	747	1.87	17.6
194	oligochaete	39	1 day	770	1.54	13.1
199	oligochaete	40	1 day	782	8.91	75.7
200		Neg. ctrl			0	0.0
201	oligochaete	25	2 m	757	1.54	14.5
202	oligochaete	26	2 m	745	5.06	45.2
203	oligochaete	27	2 m	713	8.47	81.7

Sample Name	Taxonomic group	No specimen	Storage period	Length (base pairs)	DNA concentration	RFU
204	oligochaete	28	2 m	696	11.88	136.2
205	oligochaete	29	2 m	777	3.19	26.7
206	oligochaete	30	2 m	781	1.98	16.1
207	oligochaete	31	2 m	765	1.54	13.6
208	oligochaete	32	2 m	769	0.99	8.6
209	oligochaete	33	2 m	752	3.41	31.8
210	oligochaete	34	2 m	777	0.77	6.2
211	oligochaete	35	2 m		0	0.0
212	oligochaete	36	2 m	742	5.28	54.7
213	oligochaete	37	2 m	747	3.19	30.7
214	oligochaete	38	2 m	734	1.87	19.4
215	oligochaete	39	2 m	750	4.95	49.1
220	oligochaete	40	2 m	765	1.21	10.6
221		Neg. ctrl			0	0.0
222	oligochaete	25	3 m	752	2.09	20.0
223	oligochaete	26	3 m	740	2.64	25.5
224	oligochaete	27	3 m	705	7.59	77.4
225	oligochaete	28	3 m	718	5.5	56.1
226	oligochaete	29	3 m	760	4.18	37.6
227	oligochaete	30	3 m	779	1.54	12.5
228	oligochaete	31	3 m	762	2.2	20.2
229	oligochaete	32	3 m	764	1.65	15.4
230	oligochaete	33	3 m	747	4.84	50.2
231	oligochaete	34	3 m	770	4.18	39.7
232	oligochaete	35	3 m	760	2.2	20.6
233	oligochaete	36	3 m	730	8.03	79.6
234	oligochaete	37	3 m	782	0.66	2.4
235	oligochaete	38	3 m		0	0.0
236	oligochaete	39	3 m	744	5.28	54.0
237	oligochaete	40	3 m	780	1.43	11.3
238		Neg. ctrl			0	0.0
239	oligochaete	25	4 m	740	6.27	60.8
240	oligochaete	26	4 m	727	6.16	62.2
241	oligochaete	27	4 m	720	5.72	62.0
242	oligochaete	28	4 m	730	4.07	42.8
243	oligochaete	29	4 m	775	2.09	19.3
244	oligochaete	30	4 m	754	2.42	23.5
245	oligochaete	31	4 m	761	4.18	39.4
246	oligochaete	32	4 m	771	0.55	4.7
247	oligochaete	33	4 m	758	1.54	15.0
248	oligochaete	34	4 m	792	0.55	4.5
249		Neg. ctrl			0	0.0
250	oligochaete	25	6 m	742	7.7	76.9

Sample Name	Taxonomic group	No specimen	Storage period	Length (base pairs)	DNA concentration	RFU
251	oligochaete	26	6 m	724	12.43	128.0
252	oligochaete	27	6 m	693	23.54	259.7
253	oligochaete	28	6 m	709	18.7	198.6
254	oligochaete	31	6 m	788	0.99	7.8
255		Neg. ctrl				0.0
256	chironome	41	1 day	672	44.66	531.7
257	chironome	42	1 day	680	42.24	481.5
258	chironome	43	1 day	702	29.15	323.7
259	chironome	44	1 day	716	19.91	208.7
260	chironome	45	1 day	740	7.81	77.0
261	chironome	46	1 day	729	12.98	127.6
262	chironome	47	1 day	730	10.01	90.9
263	chironome	48	1 day	747	4.51	39.1
264	chironome	49	1 day	683	35.75	410.0
265	chironome	50	1 day	683	39.82	452.4
266	chironome	51	1 day	697	30.8	345.4
267	chironome	52	1 day	725	15.62	160.8
268	chironome	53	1 day	740	10.34	100.0
269	chironome	54	1 day	737	9.9	90.9
270	chironome	55	1 day	743	3.19	28.4
271	chironome	56	1 day	732	7.7	72.9
272	chironome	57	1 day	673	44.11	508.3
273	chironome	58	1 day	698	31.68	356.5
274	chironome	59	1 day	708	25.19	275.0
275	chironome	60	1 day	733	10.89	107.1
276	chironome	61	1 day	743	5.28	47.7
277	chironome	62	1 day	746	4.84	42.8
278	chironome	63	1 day	738	5.5	50.4
279	chironome	64	1 day	745	4.29	38.0
280	chironome	41	1.5 m	687	33.55	384.0
281	chironome	42	1.5 m	713	20.79	228.6
282	chironome	43	1.5 m	737	9.79	99.3
283	chironome	44	1.5 m	750	2.42	21.8
284	chironome	45	1.5 m	747	4.29	38.9
285	chironome	46	1.5 m	763	1.21	10.2
286	chironome	47	1.5 m	766	1.87	16.3
287	chironome	48	1.5 m	769	1.65	13.4
288	chironome	49	1.5 m	702	27.06	299.4
289	chironome	50	1.5 m	708	27.61	303.3
290	chironome	51	1.5 m	728	14.85	153.0
291	chironome	52	1.5 m	746	6.82	67.1
292	chironome	53	1.5 m	735	12.54	122.5
293	chironome	54	1.5 m	746	8.14	77.6

Sample Name	Taxonomic group	No specimen	Storage period	Length (base pairs)	DNA concentration	RFU
294		Neg. ctrl			0	0.0
295	chironome	41	2 m	732	11.55	109.2
296	chironome	42	2 m	677	43.78	500.4
297	chironome	43	2 m	674	49.83	561.9
298	chironome	44	2 m	706	27.5	304.3
299	chironome	45	2 m	686	42.68	473.3
300	chironome	46	2 m	730	16.39	165.4
301	chironome	47	2 m	741	11.22	107.8
302	chironome	48	2 m	729	12.1	114.1
303	chironome	49	2 m	723	20.9	212.3
304	chironome	50	2 m	691	30.69	362.6
305	chironome	51	2 m	669	48.18	550.7
306	chironome	52	2 m	709	24.42	264.2
307	chironome	53	2 m	703	27.17	292.4
308	chironome	54	2 m	734	16.39	159.0
309	chironome	55	2 m	724	17.93	174.6
310	chironome	56	2 m	748	5.17	43.0
311	chironome	57	2 m	731	9.9	90.3
312	chironome	58	2 m	669	47.74	554.1
313	chironome	59	2 m	710	20.46	223.0
314	chironome	60	2 m	687	35.53	393.1
315	chironome	61	2 m	689	35.53	385.2
316	chironome	62	2 m	733	12.98	125.7
317	chironome	63	2 m	747	7.92	71.0
318	chironome	64	2 m	734	7.7	68.9
319		Neg. ctrl			0	0.0
320	chironome	41	3 m	670	45.98	530.4
321	chironome	42	3 m	696	29.59	328.1
322	chironome	43	3 m	679	44.55	495.4
323	chironome	44	3 m	686	39.93	435.5
324	chironome	45	3 m	697	33.22	356.3
325	chironome	46	3 m	717	22.44	221.0
326	chironome	47	3 m	736	13.53	118.6
327	chironome	48	3 m	740	11	97.5
328	chironome	49	3 m	664	50.82	585.2
329	chironome	50	3 m	695	31.79	353.5
330	chironome	51	3 m	689	38.5	418.4
331	chironome	52	3 m	704	29.15	309.9
332	chironome	53	3 m	696	33.33	353.9
333	chironome	54	3 m	699	34.21	351.7
334	chironome	55	3 m	710	27.5	275.9
335	chironome	56	3 m	744	8.91	79.4
336	chironome	57	3 m	698	28.05	308.0

Sample Name	Taxonomic group	No specimen	Storage period	Length (base pairs)	DNA concentration	RFU
337	chironome	58	3 m	691	35.75	395.3
338		Neg. ctrl			0	0.0
339	chironome	41	4 m	730	13.75	135.9
340	chironome	42	4 m	739	5.72	49.6
341	chironome	43	4 m	740	6.82	62.0
342	chironome	44	4 m	737	5.94	56.4
343	chironome	45	4 m	738	8.47	78.5
344	chironome	46	4 m	680	39.16	446.6
345	chironome	47	4 m	681	43.12	479.2
346	chironome	48	4 m	708	27.39	291.0
347	chironome	49	4 m	738	11.22	106.7
348	chironome	50	4 m	738	7.81	68.1
349	chironome	51	4 m	744	4.29	37.6
350	chironome	52	4 m	745	4.07	36.3
351	chironome	53	4 m	752	12.54	120.0
352	chironome	54	4 m	689	29.59	366.5
353	chironome	55	4 m	700	21.89	256.7
354	chironome	56	4 m	711	18.59	208.6
355	chironome	57	4 m	668	43.45	504.4
356	chironome	58	4 m	677	37.84	429.7
357	chironome	59	4 m	716	13.42	148.7
358	chironome	60	4 m	679	36.41	416.8
359	chironome	61	4 m	675	36.63	428.0
360	chironome	62	4 m	685	29.15	346.5
361	chironome	63	4 m	724	14.3	153.9
362	chironome	64	4 m	697	34.21	370.9
363		Neg. ctrl			0	0.0
364	chironome	41	6 m	764	10.56	98.6
365	chironome	42	6 m	772	7.92	71.6
366	chironome	43	6 m	766	13.86	126.6
367	chironome	44	6 m	766	9.13	81.2
368	chironome	45	6 m	690	25.19	295.1
369	chironome	46	6 m	711	21.56	241.6
370	chironome	47	6 m	683	35.09	364.7
371	chironome	48	6 m	742	15.73	159.9
372	chironome	49	6 m	731	21.67	221.4
373	chironome	50	6 m	753	15.62	148.7
374	chironome	51	6 m	752	13.75	133.0
375	chironome	52	6 m	747	17.05	164.2
376	chironome	53	6 m	718	12.54	137.4
377	chironome	54	6 m	713	16.72	186.1
378	chironome	55	6 m	727	13.86	148.2
379	chironome	56	6 m	771	2.75	25.4
380	chironome	57	6 m	754	13.53	131.3
381	chironome	58	6 m	763	10.34	96.4
382	chironome	59	6 m	767	8.69	79.0
383	chironome	60	6 m	776	5.5	46.5
384	chironome	61	6 m	721	10.78	120.5
385	chironome	62	6 m	721	17.6	195.6
386	chironome	63	6 m	759	5.83	55.9
387	chironome	64	6 m	756	11	107.7
388		Neg. ctrl			0	0.0
389	AE buffer	Neg. ctrl			0	0.0

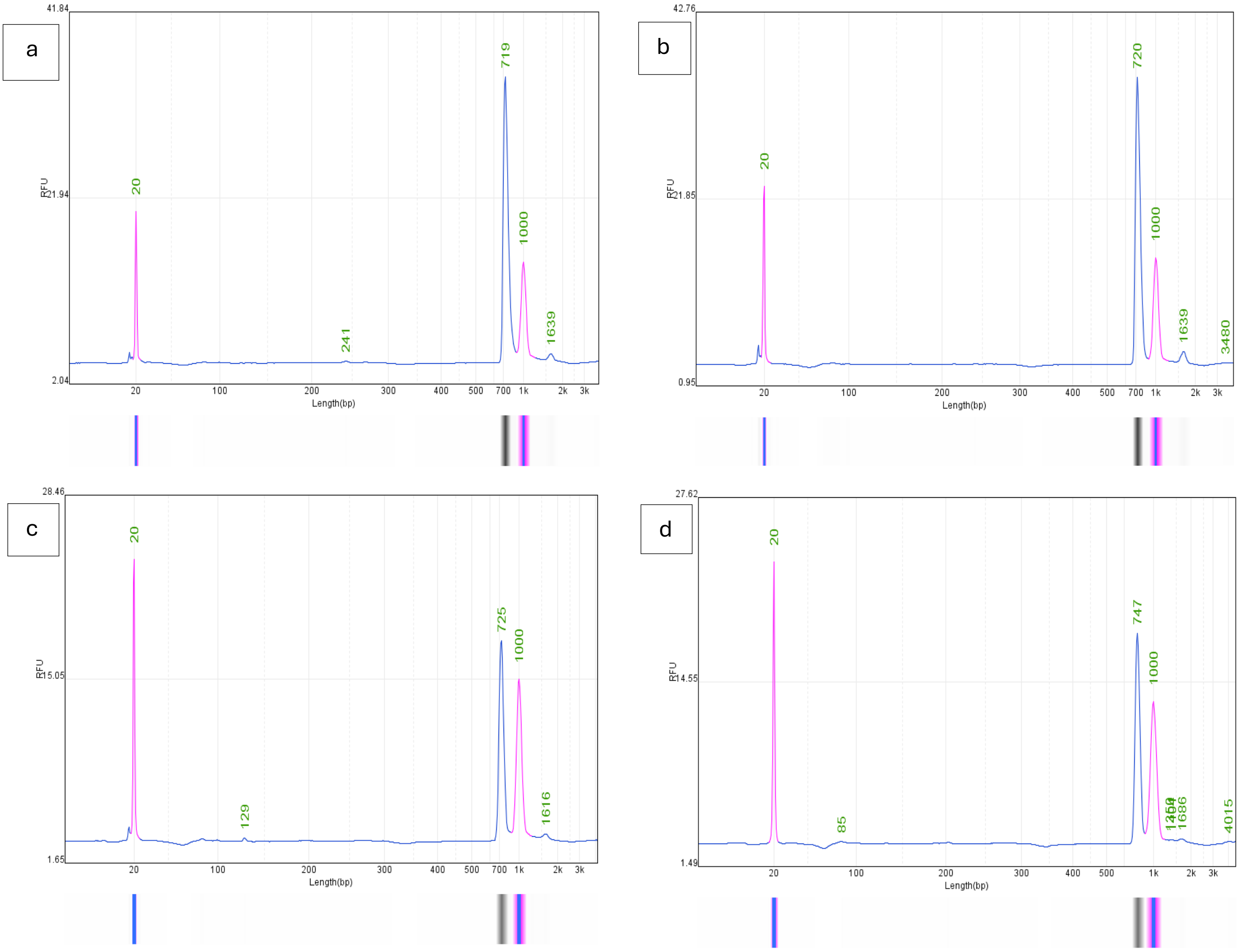

Figure S1. Representative COI amplicon profiles (electropherograms) obtained via Qsep100 capillary electrophoresis. (a) Oligochaete specimen (No 11) at 1 day of storage; (b) Same oligochaete specimen at 6 months; (c) Chironomid specimen (No 52) at 1 day; (d) Same chironomid specimen at 6 months

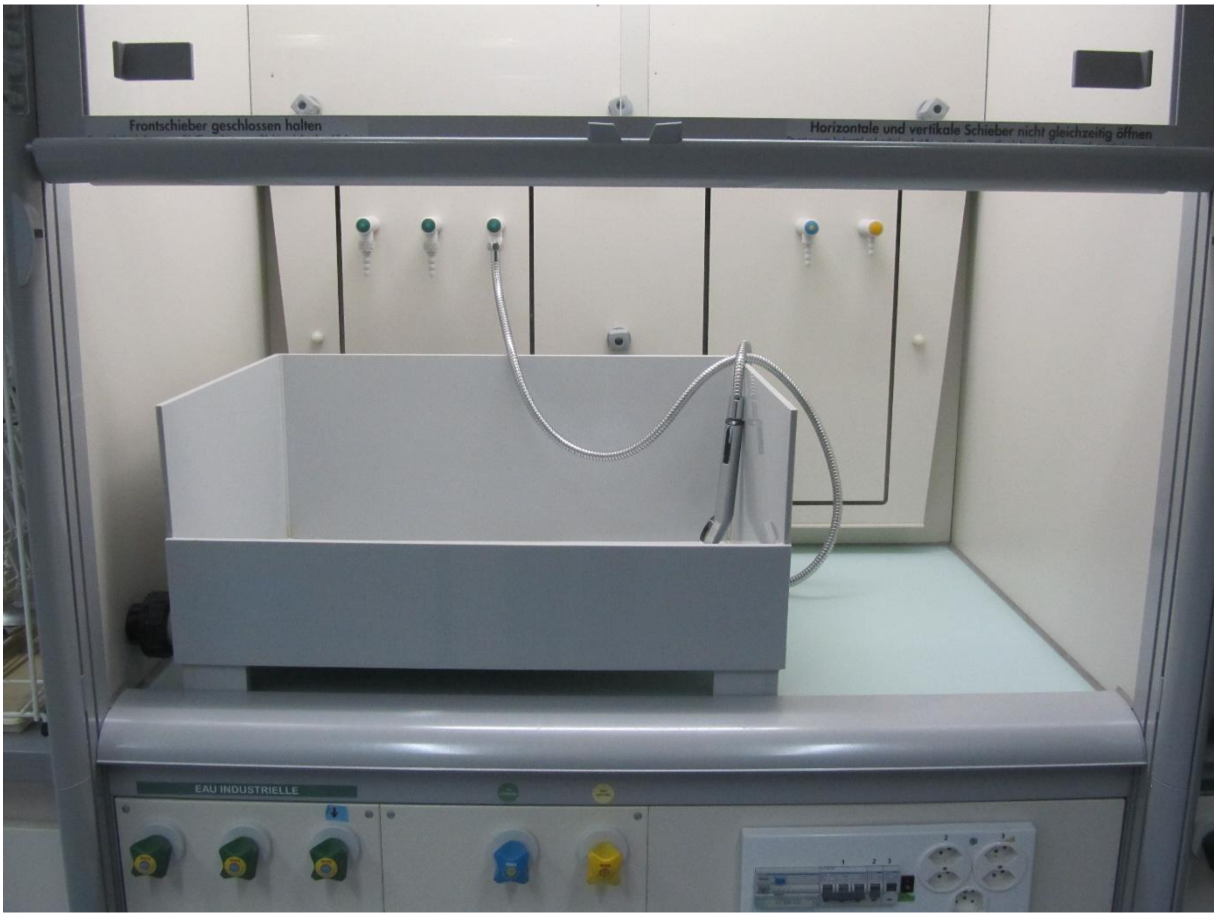

Figure S2. Photo of a sample sieving setup (consisting of a basin and showerhead) installed in a fume hood
